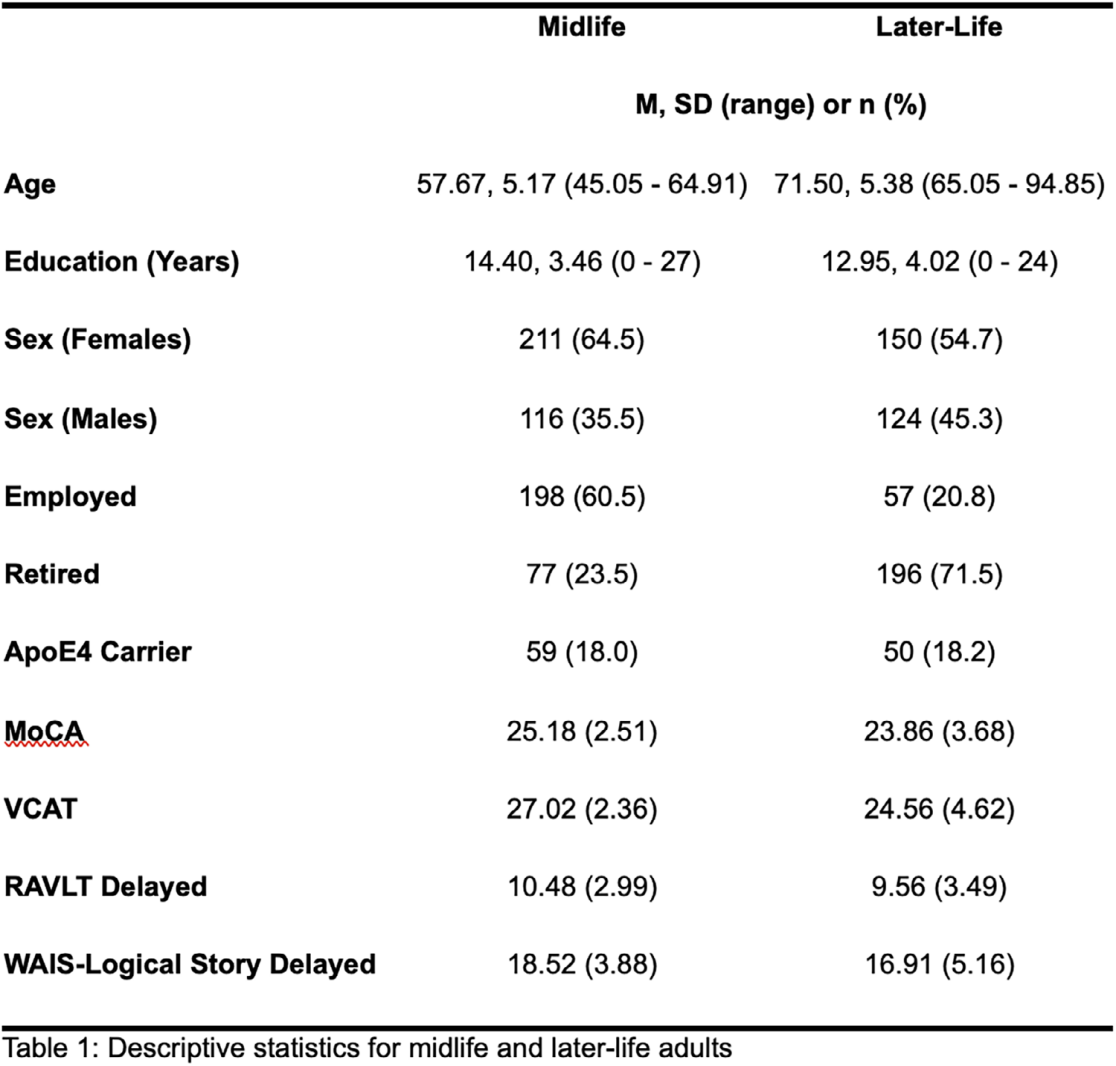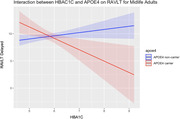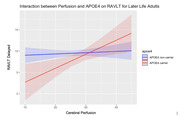# ApoE4 Status Moderates the Association between Vascular Risks, Cerebral Perfusion and Cognitive Performance in a Southeast Asian Population

**DOI:** 10.1002/alz.092285

**Published:** 2025-01-09

**Authors:** Smriti Ghildiyal, Ashwati Vipin, Gurveen Kaur Sandhu, See Ann Soo, Pricilia Tanoto, Fatin Zahra Zailan, Yi Jin Leow, Faith Phemie Hui En Lee, Shan Yao Liew, Isabelle Yu Zhen Tan, Mohammed Adnan Azam, Dilip Kumar, Chao Dang, James Xiao Yuan Chen, Nagaendran Kandiah, Dementia Research Centre (Singapore)

**Affiliations:** ^1^ Lee Kong Chian School of Medicine, Nanyang Technological University, Singapore Singapore; ^2^ Lee Kong Chian School of Medicine, Singapore Singapore; ^3^ The First Affiliated Hospital, Sun Yat‐sen University, Guangzhou China

## Abstract

**Background:**

Factors contributing to cognitive decline in adults include vascular risk factors (hypertension, hyperlipidemia, diabetes), lower education, age, Apolipoprotein E4(ApoE4) and cerebral hypoperfusion. The interplay between aging and vascular processes disrupts cerebral hemodynamics, heightening the risk of cognitive impairment and neurological disorders. Similarly, ApoE4 gene increases the risk of vascular‐related cognitive impairment and small vessel disease. The interaction between vascular risk factors, cerebral perfusion and ApoE4 carrier status on cognitive performance needs further exploration in a Southeast Asian population.

**Method:**

601 participants(Age=62.53±10.19) were recruited from the community‐based BIOCIS study at Dementia Research Centre(Singapore). Participants with a research diagnosis of Subjective Cognitive Decline(SCD), Mild Cognitive Impairment(MCI) or dementia were included and stratified into midlife (45‐65, n=327) and later‐life (>65, n=274).

Participants were administered global cognitive tests: Montreal Cognitive Assessment(MoCA), Visual Cognitive Assessment Test(VCAT), and a comprehensive neuropsychological test battery including tests for episodic memory(EM). Vascular risk factors included averaged systolic blood pressure(BP) and fasting HBA1C levels. ApoE allelic variation was determined via Taqman SNP genotyping qRT‐PCR methodology. Arterial Spin Labelling MRI data was used to quantify cerebral perfusion using the BASIL toolbox.

**Result:**

During midlife, linear regression analyses indicted elevated levels of vascular risk factors(BP and HBA1C) and positive ApoE4 carrier status were associated with poorer cognitive performance. The results demonstrated a significant interaction effect between BP and ApoE4 carrier status on global cognition: VCAT(β=‐0.031, p=0.050), MoCA (β=‐0.037, p=0.045) and between HBA1C and ApoE4 carrier status on EM scores: RAVLT delayed trial(β=‐2.68, p=0.001).

During later‐life, lowered levels of cerebral perfusion were associated with poorer cognitive performance in ApoE4 carriers only. The results found a marginally significant interaction effect between cerebral perfusion and ApoE4 carrier status on global cognition: VCAT(β=0.2286, p=0.060), and EM: RAVLT delayed (β=0.298, p=0.024), WAIS‐Logical Story (β=0.392, p=0.007). All results remained significant despite controlling for age, education, syndrome severity and consumption of medication.

**Conclusion:**

The presence of ApoE4 allele and vascular risk factors lead to a decline in global cognition and EM scores during midlife for a Southeast Asian population. ApoE4 moderates the relationship between reduced cerebral perfusion and poorer global cognition and EM scores in later‐life. Aggressively treating vascular risk during midlife, especially for ApoE4 carriers may prevent further cognitive decline during later‐life.